# Potent Antimicrobial Activity of *Aspergillus oryzae* Fermentate Against Toxigenic Strains of *Clostridioides difficile*

**DOI:** 10.3390/antibiotics14040333

**Published:** 2025-03-22

**Authors:** Ahmad Alshannaq, Morgan Henning, Jonah Dixon, Colleen Riley, Dasol Choi, Jae-Hyuk Yu, Nasia Safdar

**Affiliations:** 1 School of Medicine and Public Health, University of Wisconsin-Madison, Madison, WI 53706, USA; 2 Department of Medicine, William S. Middleton Memorial Veterans Hospital, Madison, WI 53705, USA; 3 Department of Bacteriology, University of Wisconsin-Madison, Madison, WI 53706, USA

**Keywords:** *Clostridioides difficile*, fungal extract, *Aspergillus oryzae*, fermentate

## Abstract

**Background:** *Clostridioides difficile* infection (CDI) remains a significant public health challenge in the United States, with limited treatment options currently available. **Objectives:** This study investigated the antimicrobial efficacy of a fungal-based fermentate derived from *Aspergillus oryzae*, cultivated in a proprietary food-grade medium, against toxigenic strains of *C. difficile*. **Methods and Results**: The ethyl acetate extract of *A. oryzae* fermentate (fungal extract) exhibited potent bactericidal activity, producing a significant zone of inhibition across all tested *C. difficile* strains, including hypervirulent Ribotype 027. Notably, 80% of the tested strains (four out of five) exhibited greater susceptibility to the fungal extract than to 5 µg vancomycin discs. Inner colony formation within the zone of inhibition was observed for all strains treated with vancomycin but only one strain was exposed to fungal extract. Time kill assays further confirmed the rapid bactericidal effect of the fungal extract, achieving complete *C. difficile* eradication within six hours. Mechanistic studies using scanning electron microscopy (SEM) and flow cytometry revealed that the fungal extract induced severe membrane disruption, leading to intracellular leakage and complete lysis. Flow cytometry analysis confirmed membrane depolarization and permeability loss on *C. difficile* cells. **Conclusions:** These findings highlight that the fungal extract of *A. oryzae* exhibits a promising antimicrobial activity against *C. difficile*. Future studies will focus on identifying its active components, evaluating its efficacy in vivo, and assessing its impact on gut microbiota to establish its potential clinical application in managing CDI.

## 1. Introduction

*Clostridioides difficile* is a spore-forming and toxin-producing anaerobic bacillus causing diarrhea and life-threatening colitis in nearly half a million people in the United States with a cost burden estimated at USD 5.4 billion [1,2,3]. *Clostridioides difficile* is a multi-drug-resistant organism (MDRO) that resists numerous antibiotics frequently used in clinical settings to treat bacterial infections, including aminoglycosides, lincomycin, erythromycin, clindamycin, penicillins, cephalosporins, and fluoroquinolones [4,5]. Currently, there are few therapeutic options for first-line treatment, with fidaxomicin being preferred; however, vancomycin remains the most commonly used due to its greater accessibility [6,7,8,9]. Up to 30% of patients with an initial CDI will develop a recurrence (rCDI), and up to 65% of patients who experience at least one recurrence will suffer a subsequent recurrence [8,10,11,12]. Given these challenges, there is a critical need for new antimicrobial agents to effectively combat *C. difficile* and reduce the burden of this infection.

Filamentous fungi serve as a rich resource for producing organic acids, proteins, enzymes, antibiotics, statins, and steroids [13]. Amongst these, *Aspergillus oryzae*, an edible filamentous fungus recognized by the FDA as Generally Recognized as Safe (GRAS), is extensively used in fermenting foods such as sake, miso, soy sauce, and meju [14]. In our previous work [15,16], we demonstrated the antimicrobial activity of *A. oryzae* fermentate against various aerobic Gram-positive bacterial species, including *Staphylococcus aureus* and *Listeria monocytogenes*, as well as, to a lesser extent, Gram-negative bacteria such as *Escherichia coli* and *Salmonella Typhimurium*. Additionally, the fermentate exhibited strong activity against fungal pathogens, including *Aspergillus fumigatus*, *Candida albicans*, and *Penicillium roqueforti*. We also assessed its potential toxicity using MCF-7 breast cancer cells and found that treatment with 500 µg/mL of lyophilized *A. oryzae* fermentate had no adverse effect on cell viability [15].

We studied the antimicrobial activity of ethyl acetate extract of *A. oryzae* fermentate (fungal extract) against several toxigenic *C. difficile* strains. We found that 80% of the tested strains showed greater susceptibility to the fungal extract than to 5 µg of vancomycin. The fungal extract exhibited a rapid bactericidal effect, causing severe membrane disruption and cell lysis. It induced significant depolarization and increased membrane permeability in *C. difficile* cells. These findings highlight the potential of the fungal extract as a promising candidate for developing new antimicrobials against CDI, warranting further in vitro and in vivo studies.

## 2. Results

### 2.1. The Antimicrobial Activity of the Fungal Extract

The antimicrobial activity of the fungal extract was evaluated using a disc diffusion assay and time kill assay against *C. difficile* vegetative cells. Qualitative and quantitative results estimating the antimicrobial activity of the fungal extract were obtained by observing and measuring the diameter of the zone of inhibition. The fungal extract effectively inhibited the growth of all five *C. difficile* strains, resulting in the formation of a clear zone of inhibition. The positive control (5 µg vancomycin) showed a potent zone of inhibition as well. However, zones with inner colonies were reported in all strains tested against 5 µg vancomycin discs. In contrast, this scenario was noted for only one strain ORLVS 052 that has the binary toxin gene (*cdtB*).

**Figure 1:**
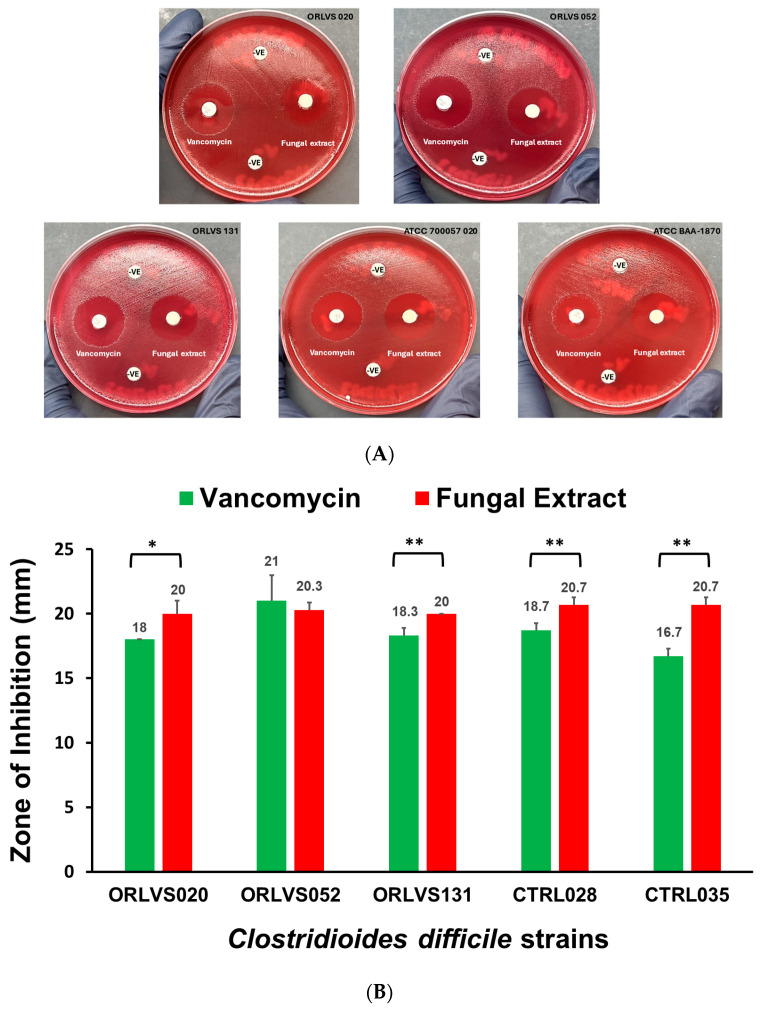

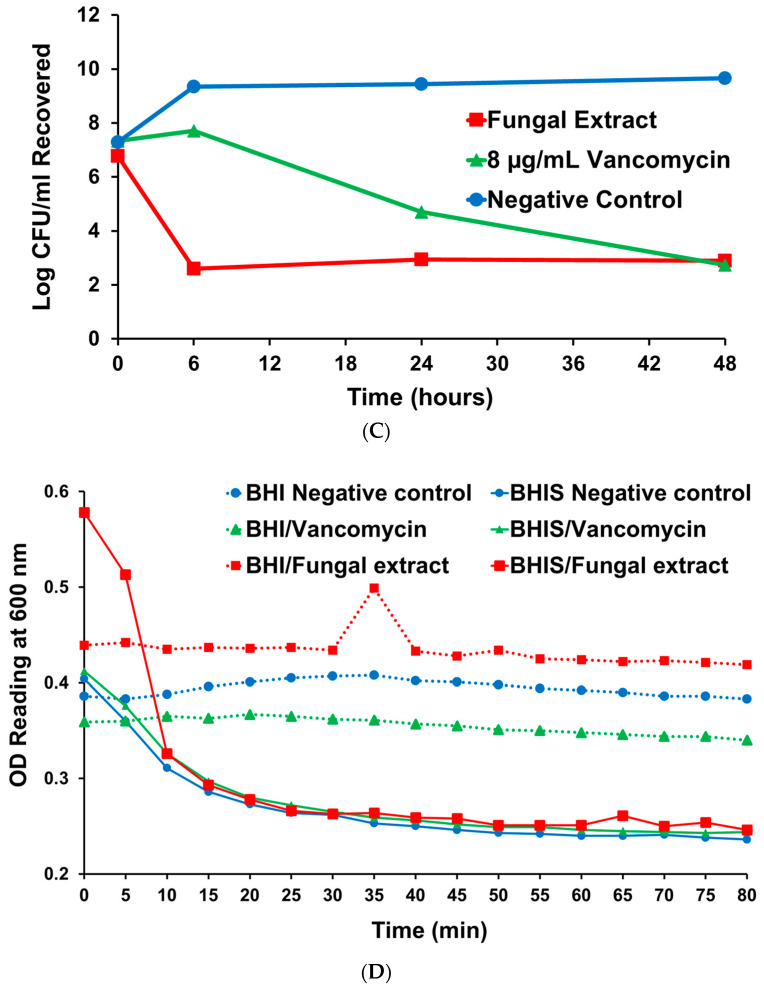
**Antimicrobial Activity of the fungal extract.** (**A**) BBA plates showing the zone of inhibition for fungal extract (right) and 5 µg vancomycin (left) against five *C. difficile* strains as tested by agar disc assay. Discs were loaded with 100 µL of fungal extract, dried, and placed onto the agar. Negative controls (-VE) included discs loaded with 100 µL of fungal-free broth, processed similarly to the fungal extract (top), and a disc loaded with methanol, dried before use (bottom). (**B**) Comparative zone of inhibition diameter (mm) for the fungal extract and 5 µg vancomycin against five *C. difficile* strains. Data are presented as mean ± SD. Data were considered statistically significant when * *p* ≤ 0.05 or ** *p* ≤ 0.01. (**C**) The time kill assay of fungal extract and vancomycin (8 µg) against hypervirulent *C. difficile* strain Ribotype 027 (ATCC BAA-1870). (**D**) The spore germination assay of the fungal extract and vancomycin (8 µg) against hypervirulent *C. difficile* strain Ribotype 027 (ATCC BAA-1870). BHI is a brain heart infusion broth without supplements, while BHIS includes supplements.

No zone of inhibition was observed for the control, in the top and bottom discs (Figure 1A). The diameter of the zone of inhibition ranged from 16 to 21 mm and 20–21 mm for vancomycin and the fungal extract, respectively. The fungal extract exhibits significant antimicrobial activity when compared to vancomycin (5 µg) toward 80% *C. difficile* isolates. The *C. difficile* hypervirulent strain Ribotype 027 (ATCC BAA-1870) was the most susceptible to the fungal extract (20.7 mm) but the least susceptible to 5 µg vancomycin (16.6) mm (Figure 1B). No detectable growth of *C. difficile* was observed with the fungal extract at 6 h of incubation. More than a 4-log_10_ reduction in bacterial growth was observed, indicating that the fungal extract has excellent bactericidal activity, defined as ≥3-log_10_ bacterial growth reduction against *C. difficile.* In contrast, *C. difficile* cell growth was observed at 24 h for 8 µg vancomycin (positive control) (Figure 1C). The potential of the fungal extract to inhibit *C. difficile* spore germination was assessed. This critical process facilitates the transformation of dormant, resistant spores into metabolically active vegetative cells. In the presence of spore germinant, neither fungal extract nor vancomycin were able to inhibit spore germination of *C. difficile* (Figure 1D).

### 2.2. Mechanism of Action

**Figure 2:**
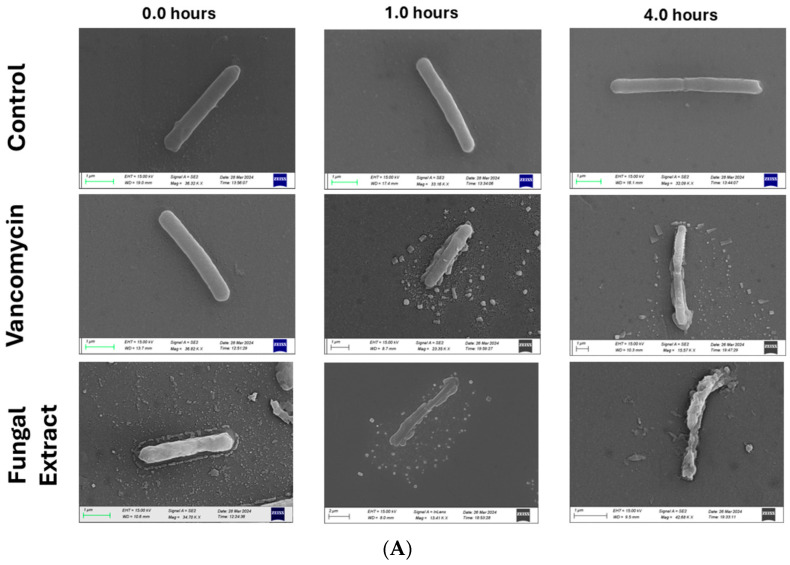

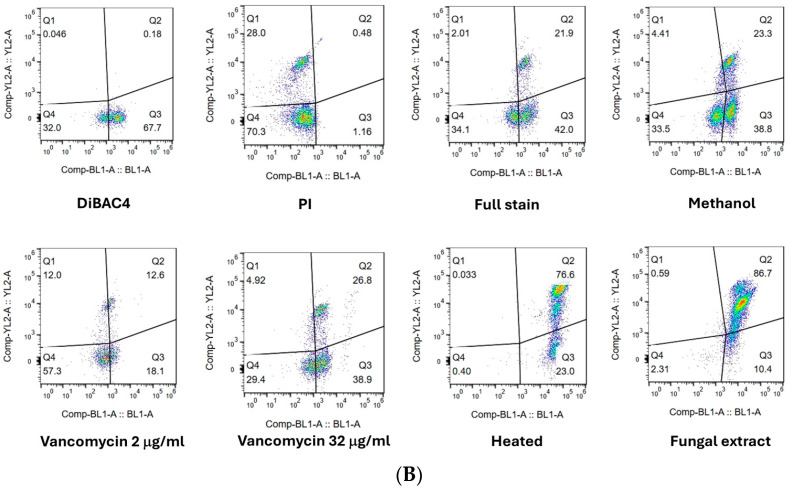
The mechanism of action of the fungal extract. (**A**) Scanning electron microscopy (SEM) images of *C. difficile* cells treated with the fungal extract, 8 µg vancomycin, and deionized (DI) water as a control. The images were captured at 0, 1, and 4 h to assess morphological changes over time. (**B**) Fluorescence dot plots of *C. difficile* stained with PI and DiBAC4. The quadrants are categorized as follows: Q1 (High PI/Low DiBAC4), Q2 (High PI/High DiBAC4), Q3 (Low PI/Low DiBAC4), and Q4 (Low PI/High DiBAC4).

To explore the mechanism of action of the fungal extract on *C. difficile* vegetative cells, scanning electron microscopy (SEM) was used to visualize potential morphological changes in the bacterial cell structure. As shown in Figure 2A, control cells treated with deionized (DI) water remained intact over a 4 h imaging period. In contrast, *C. difficile* cells treated with the fungal extract exhibited progressive leakage of intracellular components, ultimately leading to complete cell destruction after 4 h. A similar effect was observed in bacterial cells treated with vancomycin (Figure 2A). Along with SEM imaging, flow cytometry was employed to investigate the effects of the fungal extract on *C. difficile* membrane permeability and potential. To achieve this, two fluorescent dyes were used—propidium iodide (PI) to assess membrane integrity and DiBAC4 to evaluate changes in membrane potential—providing insight into the bactericidal mechanism of the fermentate. The dot plots of untreated cells (PI, DiBAC4, full stain, and methanol) showed that most of the untreated *C*. *difficile* cells were not stained with either PI or DiBAC4 (Q2) indicating no cell damage. The dot plot of *C. difficile* cells treated with the fungal extract exhibits high fluorescence, indicating dual staining with both PI and DiBAC4 (Q2). This suggests that the cells have undergone late-stage damage, characterized by both membrane permeability loss and depolarization. The same scenario played out with the heat-treated group (Q2). The dot plots of both vancomycin-treated groups were not stained PI and DiBAC4 (Q2) indicating no cell damage (Figure 2B).

## 3. Discussion

Currently, oral vancomycin and fidaxomicin are the mainstays for the treatment of initial CDI, with vancomycin being more commonly used due to its greater accessibility [6,9]. However, their high cost and impact on the gut microbiome, especially for vancomycin, pose significant challenges [17,18]. Given these limitations, the urgent development of a new generation of therapeutics is critical to address the growing need for effective and accessible CDI treatment options.

The findings of this study demonstrate the potent antimicrobial activity of *A. oryzae* fungal extract against *C. difficile*, highlighting its potential as an effective bactericidal agent. The disc diffusion assay revealed that the fungal extract produced a significant zone of inhibition against all *C. difficile* strains including non-toxigenic, clinical isolates, and hypervirulent strains such as Ribotype 027. There are significant differences in the diameter of the zone of inhibition between the fungal extract and vancomycin (5 µg) in 80% of tested strains (4 out of 5). Notably, the hypervirulent *C. difficile* strain Ribotype 027 (ATCC BAA-1870) exhibited greater susceptibility to the fungal extract than to vancomycin, suggesting that the *A. oryzae* fungal extract may target critical bacterial structures or processes distinct from those affected by vancomycin. Inhibition zones with inner colonies have been reported for all *C. difficile* strains tested against vancomycin, but only present for one strain tested against the fungal extract. The presence of inner colonies within the zone of inhibition suggests potential heteroresistance, tolerance, or adaptive resistance among *C. difficile* populations [19]. These colonies may represent subpopulations with partial resistance, persisted cells, or bacteria capable of inactivating the antimicrobial agent [20]. Alternatively, slow-growing variants or experimental factors could contribute to their emergence. Further analysis, including susceptibility retesting, genetic characterization, and enzyme activity assays, is necessary to determine the underlying mechanism. Understanding these factors is crucial for accurately assessing antimicrobial efficacy and resistance development for both vancomycin and *A. oryzae* fermentate.

The time kill assay further validated the potency of *A. oryzae* fungal extract, achieving complete eradication of *C. difficile* within six hours. In contrast, *C. difficile* continued to grow for 24 h when treated with vancomycin at 8 µg/mL. Despite its efficacy against vegetative cells, neither the fungal extract nor vancomycin inhibited *C. difficile* spore germination, indicating that the extract does not interfere with the early stages of spore outgrowth. The spore core is encased by multiple protective layers, including the inner membrane, cell wall, cortex, outer membrane, spore coats, and exosporium. These layers form a permeability barrier that contributes to spore resistance by preventing the entry of DNA-damaging compounds [3].

To elucidate the mechanism of action, scanning electron microscopy (SEM) and flow cytometry were employed to assess cellular morphology and membrane integrity. SEM imaging revealed extensive cellular damage in *C. difficile* cells treated with the fungal extract, characterized by intracellular leakage and complete cell lysis after four hours. This morphological disruption closely paralleled the effects observed with vancomycin, reinforcing the bactericidal nature of the extract. Flow cytometry analysis provided additional mechanistic insights, demonstrating that fungal extract induces both membrane depolarization and loss of membrane integrity, as evidenced by dual staining with propidium iodide (PI) and DiBAC4. The significant shift in fluorescence intensity, particularly in quadrant Q2, indicates late-stage bacterial damage characterized by extensive membrane disruption. In contrast, vancomycin-treated cells did not show the same staining pattern, suggesting that at the tested concentrations, vancomycin did not induce sufficient membrane permeability loss to enhance dye uptake. Given that vancomycin primarily targets peptidoglycan synthesis rather than directly disrupting membrane integrity, it is possible that higher concentrations (>32 µg/mL) may be required to produce similar effects.

These findings suggest that *A. oryzae* fungal extract represents a promising natural antimicrobial with potent activity against *C. difficile*. Its ability to induce membrane damage and bacterial lysis sets it apart from conventional antibiotics, which may act more slowly or through different mechanisms. However, additional studies are needed to fully characterize the mechanism of action and its active components, to explore its potential synergy with existing therapies, and to assess its effectiveness in vivo models of *C. difficile* infection. Furthermore, understanding the impact of *A. oryzae* fungal extract on the gut microbiota will be essential to evaluate its therapeutic potential and safety in clinical applications.

## 4. Materials and Methods

### 4.1. Fungal Strain and Fermentate Production

*A. oryzae* NRRL 3483 was used in this study to produce the fermentate. *A. oryzae* was cultured on potato dextrose agar (PDA) for 4 days at 30 °C. Asexual spores were harvested from individual cultures on PDA using sterile 0.1% Tween-80 solution and counted using a hemocytometer. Spore numbers were adjusted to 5 × 10^7^ conidia/mL with sterile deionized water (DI) water. The fungal spore suspension was stored at 4 °C and used within two weeks of preparation. To produce the fermentate, *A. oryzae* NRRL 3483 was inoculated at a final concentration of 5 × 10^5^ conidia/mL into 250 mL Erlenmeyer flasks containing 150 mL of liquid culture medium and incubated for 6 days at 25 ± 2 °C with shaking at 220 rpm. The liquid culture medium contains 17.0 g pancreatic digest of casein, 3.0 g papain digest of soybean, 2.5 g dextrose, 5.0 g sodium chloride, and 2.5 g dipotassium phosphate in 1000 mL distilled water. At the end of the incubation period, *A. oryzae* mycelia were separated from the culture broth by filtration through four layers of Miracloth (MilliporeSigma, Burlington, MA, USA), and the sterile cell-free culture fermentate, *A. oryzae* fermentate (AOF), was obtained by filtering through a 0.22 mm PES membrane filter unit (Thermo Scientific, Waltham, MA, USA). As a control group, a liquid culture medium with no fungus was treated similarly. *A. oryzae* fermentate was stored in the fridge at 5 °C throughout the experiments. “*A. oryzae* fermentate” refers to the entire cell-free culture supernatant obtained directly from the fermentation of *Aspergillus oryzae*, without any solvent-based extraction.

Preparation of the fungal extract: 20 mL of *A. oryzae* fermentate (AOF) was mixed with an equal volume of ethyl acetate (EA) (*v*/*v*) in a 50 mL conical tube and incubated overnight at 25 °C with shaking at 150 rpm. Then, the mixture was centrifuged at 5000 rpm and the top organic ethyl acetate layer was transferred to a new tube and dried under gentle airflow, reconstituted in 1.0 mL of methanol (Thermo Fisher Scientific, Waltham, MA, USA), and filtered using a sterile 0.45 μm PES membrane filter unit (Thermo Fisher Scientific, USA). The resulting methanol solution, referred to as the “fungal extract”, was stored at 5 °C and utilized throughout the study. “The fungal extract” refers to the ethyl acetate extract derived from the fermentate, which is a more purified or concentrated form of the bioactive compounds present in the fermentate.

### 4.2. Bacterial Strains and Culture Conditions

Five *C. difficile* strains were used in this study: a non-toxigenic strain (ATCC 700057), a toxigenic hypervirulent ribotype 027 strain (ATCC BAA-1870), and three clinical toxigenic isolates: a tcdA+ tcdB+ cdtB− isolate (ORLVS 020), a tcda+ tcdB+ cdtB+ isolate (ORLVS 052), and a tcdA− tcdb+ cdtB− isolate (ORLVS 131). All strains and isolates were cultured from frozen glycerol stocks −80 °C either on pre-reduced Tryptone Soya Agar (TSA) with 5% sheep blood (Thermo Scientific, USA) or in pre-reduced brain heart infusion broth (DOT Scientific, Burton, MI, USA) supplemented with 5 g/L yeast extract (Thermo Fisher, Waltham, MA, USA), 1% L-cysteine (Sigma-Aldrich, St. Louis, MO, USA), and 0.1% sodium taurocholate (Sigma-Aldrich, USA) (BHIS). The cultures were incubated at 37 °C for 24–48 h under anaerobic conditions prior to use.

### 4.3. Antimicrobial Activity: Agar Disc Diffusion Assay

Agar disc diffusion assay was used to assess the antimicrobial activity of the fungal extract against all five *C. difficile* strains and compared with 5 µg vancomycin (BD Diagnostic, Franklin Lakes, NJ, USA) according to the Antimicrobial Susceptibility Testing Standards outlined by the Clinical and Laboratory Standards Institute [21] and method for antimicrobial susceptibility testing of anaerobic bacteria [22], with some modifications. Briefly, 100 mL of the fungal extract was loaded onto sterile blank 6 mm discs (BD, Franklin Lakes, NJ, USA) and air dried under a Biological Safety Cabinet (BSC). A sterile blank 6 mm disc was loaded with methanol (Sigma, St. Louis, MO, USA) and dried and served as a control. To standardize the inoculum density, single colonies of *C. difficile* were picked, and the turbidity was adjusted by pre-reduced Phosphate-buffered saline (PBS) to match a 0.5 McFarland standard. This results in a bacterial suspension containing approximately ∼10^7^ colony-forming units (cfu)/mL. The bacterial suspension density was confirmed to be 0.08–0.1 using a spectrophotometer at a 600 nm absorbance.

A cotton applicator was submerged into the PBS bacterial suspension and then streaked onto Brucella Blood Agar (BBA, Fisher Scientific, Waltham, MA, USA). BBA plates are supplemented with Hemin and Vitamin K (BBA, Fisher Scientific) and were reduced for 24 h in the anaerobic chamber to ensure the removal of residual oxygen [23]. Then, all discs were placed on the surface of BBA plates and incubated anaerobically at 37 °C for 48 h. This process was completed in triplicate. After the incubation period, the colony-free zone of inhibitions was measured in millimeters (mm) using a ruler and recorded.

### 4.4. Time Kill Assay

A time kill assay was conducted against the hypervirulent *C. difficile* ATCC BAA-1870 to assess whether the fungal extract exhibits bactericidal or bacteriostatic activity [24,25]. Log-phase cultures of *C. difficile* (10^8^ cfu/mL) were incubated at 37 °C in a falcon tube with the fungal extract, vancomycin at a final concentration of 8 µg/mL and negative control in pre-reduced brain heart infusion broth (BHIS) supplemented with 5 g/L yeast extract + 1% L-cysteine + 0.1% taurocholate. One mL of the fungal extract was evaporated in a 5 mL falcon tube and used in this experiment. Sterile BHIS with no fungal extract or vancomycin was used as a negative control. Aliquots (20 µL) were taken from each treatment at specific time points of incubation (0 h, 6 h, 24 h, and 48 h), diluted, and plated onto pre-reduced BBA agar plates to determine the viable bacterial count. The experiment was performed in triplicate. Each plate was counted three times by two separate people using a tally counter. The numbers were then averaged and CFUs were determined for each plate. With this method, the threshold of detection was 100 CFU/mL. Bactericidal activity was defined by a ≥3-log10 reduction in colonies compared to the starting inoculum.

### 4.5. Scanning Electron Microscope (SEM)

Scanning electron microscopy (SEM) was used to visualize the effects of the fungal extract on the cell wall of *C. difficile* ATCC BAA-1870 [26]. To prepare the cells for imaging, 200 μL of the fungal extract was added to a microplate and left to evaporate in sterile conditions. An equal volume of BHIS was added to the wells to rehydrate the fungal extract. Also, 10 μL of sterile DI water and 500 ug/mL vancomycin solution were added to wells containing 200 μL of BHIS to prepare the negative and positive controls, respectively. To each well, 5 μL of *C. difficile* broth culture was added, pipet mixed, and incubated at 37 °C under anaerobic conditions. Samples were collected from each time point (0 h, 1 h, and 4 h) in microcentrifuge tubes, centrifuged at 10,000× *g* for 10 min, and washed with 1 mL of PBS. Samples were centrifuged again at 10,000× *g* for 10 min, resuspended in 1 mL of 4% paraformaldehyde, and incubated for 1 h at room temperature. Samples were centrifuged one more time at 10,000× *g* for 10 min and washed twice with DI water before being resuspended in 100 μL of sterile DI water. Approximately 40 μL of the suspension was pipetted onto glass microscope slides and allowed to dry in sterile conditions. Samples were transferred to the Nanoscale Imaging and Analysis Center for coating and scanning electron microscopy. The slides were coated with gold for 30 s at 80 mA in the Leica ACE600 sputtering machine (Leica Camera, Wetzlar, Germany). The slides containing the samples were affixed to the stage of the Zeiss GeminiSEM 450 (ZEISS, Jena, Germany) with copper tape before being inserted into the chamber for imaging. Samples were imaged and analyzed with the Zeiss SmartSEM software. Samples were imaged using the SE2 electron source at working distances between 5.5 and 10.5 mm and with beam voltages ranging from 5 kV to 15 kV.

### 4.6. Flow Cytometry

Flow cytometry was used to investigate the effects of the fungal extract on the permeability and membrane potential of the hypervirulent *C. difficile* strain ATCC BAA-1870 (ribotype 27). Membrane permeability and potential were assessed using propidium iodide (PI) and bis-(1,3-dibutylbarbituric acid) trimethine oxonol (DiBAC4) (Invitrogen, Carlsbad, CA, USA) [27]. A pre-culture of *C. difficile* ATCC BAA-1870 was inoculated into BHI broth and incubated for 18–24 h at 37 °C under anaerobic conditions. After incubation, the bacterial cells were harvested by centrifuging at 4500 rpm for 5 min. The resulting pellets were resuspended in BHI broth to an optical density (OD_600_) of 0.18–0.22.

The fungal extract was added to 1.5 mL microcentrifuge tubes and then dried. An equal volume of adjusted broth was added to rehydrate the samples. Vancomycin was tested at concentrations of 32 µg/μL and 2 µg/μL, and a positive control was created by heating a sample at 100 °C for 30 min. All samples were centrifuged at 4500 rpm for 5 min, and the pellets were washed with 1× phosphate-buffered saline (PBS). The pellets were then resuspended in PBS containing 10 μg/mL DiBAC4 and incubated at room temperature for 10 min. Following this incubation, the mixtures were centrifuged again at 4500 rpm for 10 min. The pellets were washed with PBS and resuspended in 500 μL of PBS containing 5 μg/mL PI. Flow cytometric analyses were performed using a ThermoFisher Attune NxT with 100,000 events collected for each sample (ThermoFisher, USA). The data were analyzed and plotted as histograms of fluorescence events against fluorescence intensity using FlowJo version 10.10.0 (BD, USA).

### 4.7. Spore Germination Assay

We conducted this assay to evaluate whether the fungal extract can inhibit *Clostridioides difficile* spore germination [28]. The hypervirulent *C. difficile* strain ATCC BAA-1870 was used for this assay. Spores were prepared and purified as described previously (31), and spore stocks were stored at −20 °C until use. About 10^7^ *C. difficile* spores were suspended in 1 mL brain heart infusion (BHI) broth with or without germinants (5 g/L yeast extract, 1% L-cysteine, and 0.1% taurocholate). Using a sterile 24-well flat bottom plate (Costar, Washington, DC, USA), 0.5 mL of the fungal extract was evaporated and then reconstituted with 1 mL of BHI with and without the supplements. In total, 8 µg/mL of vancomycin was included as a positive control, with and without supplements. As a negative control, we used BHI only with and without supplements. The spore suspension was heated at 65 °C for 20 min prior to the assay run to kill vegetative cells within the suspension. After setting all treatment groups, 1 mL of BHI with/without supplements was added and then *C. difficile* spore was swiftly added. Then, the absorbance was measured on Synergy H1 Microplate Reader at 600 nm for a time point of T0. Then, the OD_600_ was measured every 5 min for 80 min. The absorbance data were then transferred and plotted into an Excel sheet.

### 4.8. Statistical Analysis

Antimicrobial activity results are expressed as the mean ± standard deviation (SD). T-tests were used to compare the diameter of the zone of inhibition between the vancomycin-treated group and the fungal extract-treated group for each *C. difficile* strain. A *p*-value ≤ 0.05 was considered statistically significant.

## 5. Conclusions

In conclusion, *A. oryzae* fungal extract exhibits strong bactericidal activity against *C. difficile*, disrupting membrane integrity and leading to rapid bacterial death. These findings highlight its potential as a novel antimicrobial agent for combating *C. difficile* infections. However, further studies are needed to fully elucidate the mechanism of action and identify active components. Future studies will focus on evaluating its efficacy in vivo and assessing its impact on gut microbiota to establish its potential clinical application. Understanding the extract’s safety profile and therapeutic potential through comprehensive in vivo testing is essential for advancing its development as an effective treatment for CDI.

## Data Availability

The original contributions presented in this study are included in the article. Further inquiries can be directed to the corresponding author.
